# Association of Irregular Menstruation and Sleep Duration With Depression Symptoms in Women of Childbearing Age

**DOI:** 10.7759/cureus.86313

**Published:** 2025-06-18

**Authors:** Wenjing Xiong, Fengtai Liu, Zhiheng Wang, Zheng Ren, Meng Zhang, Wenhai Sui

**Affiliations:** 1 Department of Cardiology, Qilu Hospital of Shandong University, Jinan, CHN; 2 Department of Radiation Oncology, Qilu Hospital of Shandong University, Jinan, CHN; 3 Department of Hematology, Qilu Hospital of Shandong University, Jinan, CHN; 4 School of Politics and Public Administration, Guangxi Normal University, Guilin, CHN

**Keywords:** depression symptoms, irregular menstruation, nhanes, sleep duration, women of childbearing age

## Abstract

Introduction

With the decline of the fertility rate, women of childbearing age are playing an increasingly important role in social development, and paying attention to their physical and mental health is of great significance. We aimed to explore the association of irregular menstruation and sleep duration with depression symptoms in women of childbearing age.

Methods

We collected data from the National Health and Nutrition Examination Survey (NHANES) from 2013 to 2018. Irregular menstruation and sleep duration were derived from questions, and depression symptoms were assessed by questionnaire data. Generalized additive model (GAM), threshold effect analysis, and binary logistic regression were used in this study.

Results

A total of 3141 participants aged 18-45 years were enrolled in the study, and 9.55% had depression symptoms. Both irregular menstruation (OR=1.595, (95% CI: 1.065-2.389)) and sleep duration (OR=0.872, (95% CI: 0.774-0.983)) were associated with depression symptoms after adjusting for all covariates. GAM and threshold effect analysis showed a U-shaped relationship between sleep duration and depression symptoms, and the inflection point of sleep duration was nine hours. An interaction of irregular menstruation and sleep duration on depression symptoms was found (OR=0.736, (95% CI: 0.554-0.977), P=0.035).

Conclusions

Irregular menstruation and sleep duration were associated with depression symptoms, and there was an interaction of irregular menstruation and sleep duration on depression symptoms in women of childbearing age. These findings may help women of childbearing age to be aware of the negative impact of irregular menstruation and improper sleep duration on depression symptoms and might provide potential targets for intervention.

## Introduction

Depression symptoms, a major global public health concern, are expected to be the most significant cause of disease burden by 2030 [1]. A national survey of US adults indicated that the prevalence of depression symptoms is 9.3% in women and 5.6% in men; this gender difference starts from adolescence and lasts until midlife, approaching the period of women's childbearing age [2]. The childbearing period is a new stage of status transition both in sociality and family, which may grant women a new name of "mother," and women of childbearing age may suffer from different types and degrees of stress and unhealthy emotions [3]. It is imperative to focus on depression symptoms in women of childbearing age. However, previous studies on depression symptoms in women have focused on the general population or specific phases, such as antenatal and postnatal periods [4], with limited studies on women of childbearing age.

The menstrual cycle, commonly defined as a vital sign by clinicians, is an important indicator for identifying potential health concerns and reproductive health issues in women [5]. By the sixth year after menarche, approximately at age 18 in women, a normal menstrual cycle length (28±7 days, lasting three to seven days) will be established [5]. Irregular menstruation (including too long or too short menstrual cycle length, amenorrhea, and so on), a common complaint among women of childbearing age, may signal underlying health imbalances [6]. A study indicated that 5%-35% of women of childbearing age may experience irregular menstruation [7]. Irregular menstruation is a risk factor for various mental illnesses [7], and prior existing studies have explored the relationship between menstrual irregularities and depression symptoms in adolescents [8]; however, less is known about the relationship in women of childbearing age.

The appropriate sleep duration recommended by the National Sleep Foundation is seven to nine hours per day for adults aged 26-64 and seven to eight hours for adults over 65 [9]. Numerous studies have investigated the relationship between sleep duration and depression, but the results have been controversial. Some researchers demonstrated that short sleep duration was related to depression [10], while other studies supported that both short and long sleep durations were associated with depression [11]. Prior studies on the relationship between sleep duration and depression have mainly focused on the elderly population or disease populations [12], with limited research on women of childbearing age. Furthermore, the current study aims to explore the interaction of irregular menstruation and sleep duration with depression symptoms and provide potential targets for intervention.

In summary, prior studies on the relationship between irregular menstruation and depression symptoms have mainly focused on other populations; less is known about the relationship in women of childbearing age. In addition, the results of the association between sleep duration and depression symptoms remain controversial. We further explored the interaction of irregular menstruation and sleep duration on depression symptoms, which is the first study to examine their interaction in a nationally representative sample. Therefore, we hypothesized that (1) irregular menstruation is associated with higher odds of depression symptoms in women of childbearing age; (2) both short and long sleep durations are associated with higher odds of depression symptoms in women of childbearing age; and (3) there exists an interaction of irregular menstruation and sleep duration on depression symptoms in women of childbearing age.

## Materials and methods

Study design

The study was a cross-sectional study based on the National Health and Nutrition Examination Survey (NHANES). The NHANES, conducted by the Centers for Disease Control and Prevention, is a complex, multi-stage, probability sampling design survey of the non-institutionalized civilian population in the US. Details of the survey are available at the website: https://www.cdc.gov/nchs/nhanes/index.htm.

Study population

**Figure 1:**
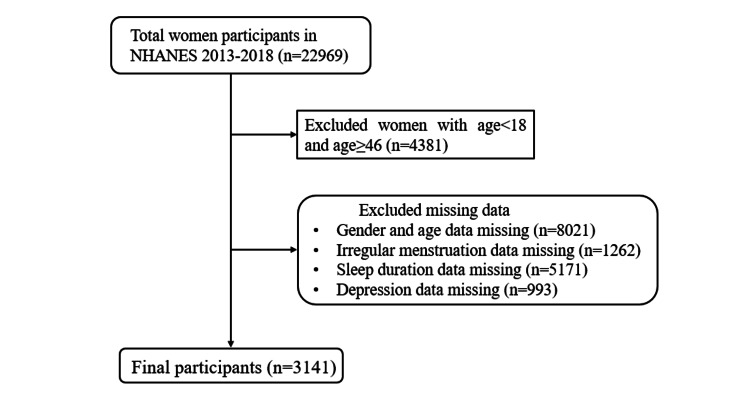
Flowchart of participants' inclusion

We obtained data on women of childbearing age (18-45 years) [13] from three cycles of NHANES: 2013-2014, 2015-2016, and 2017-2018. A total of 22,969 women participants were selected, and participants with the following criteria were excluded: (1) age < 18 and ≥ 46 years; (2) participants with missing data on depression, sleep duration, and irregular menstruation. Finally, 3141 women were included in the study (Figure 1).

Outcome variables

The Patient Health Questionnaire (PHQ-9) scale is a nine-item self-report assessment tool used to evaluate depression symptoms in participants. A score of 0-3 was given from "not at all" to "nearly every day." The total score is obtained by adding up the scores of nine items (range: 0-27) for depression symptoms. A score of 10 or more (PHQ≥10) indicates the presence of depression symptoms. The Cronbach's alpha for the PHQ-9 was 0.844 in this study.

Exposure variables

Sleep duration was obtained from a self-reported question. For the 2013-2016 cycle, participants were asked, "How much sleep do you usually get at night on weekdays or workdays (hours)?" For the 2017-2018 cycle, the question was phrased as "Sleep hours (weekdays or workdays)."

Irregular menstruation was assessed by the following question: "Have you had at least one menstrual period in the past 12 months? (Please do not include bleedings caused by medical conditions, hormone therapy, or surgeries)." Participants who replied "No" were classified as having irregular menstruation.

Confounding variables

Various confounding variables were carefully evaluated, and variables with missing values exceeding 10% were not measured. Confounding variables included age, race (non-Hispanic White/other), body mass index (BMI), smoking status, drinking status, hypertension, diabetes, and age at menarche.

The smoking status was divided into non-smoker, former smoker, and current smoker based on responses to questions regarding lifetime cigarette use, current smoking habits, and recent smoking frequency: "Have you smoked at least 100 cigarettes in your entire life?" "Do you now smoke cigarettes?" and "During the past 30 days, on the days that you smoked, about how many cigarettes did you smoke per day?" [14].

Based on responses to questions about lifetime alcohol consumption and recent drinking patterns, "Have you had at least 12 drinks of any type of alcoholic beverage?" "In your entire life, have you had at least 12 drinks of any type of alcoholic beverage?" "In the past 12 months, on those days that you drank alcoholic beverages, on average, how many drinks did you have?" and "Ever had a drink of any kind of alcohol?" The drinking status was divided into non-drinker, former drinker, and current drinker [14].

Hypertension and diabetes were assessed based on whether participants had been diagnosed by a healthcare professional, with responses being either "Yes" or "No": "Have you ever been told by a doctor or other health professional that you had hypertension or high blood pressure?" and "Have you ever been told by a doctor or health professional that you had diabetes or sugar diabetes?"

Statistical analysis

Given the complicated sample design of the NHANES, all analyses were performed using the correct NHANES sampling weights. Continuous variables were summarized as median and interquartile range (non-normal distribution), and a Mann-Whitney U test was conducted for the comparison. For categorical variables, data were presented as percentages, and a chi-square test (χ² test) was used for the comparison. A generalized additive model (GAM) and threshold effect analysis were generated to investigate the nonlinear relationship between sleep duration and depression, and the Wald chi-square test [15] was conducted to examine the nonlinearity. Binary logistic regression was performed to evaluate the relationships of irregular menstruation and sleep duration with depression symptoms under the adjustment for all covariates, and odds ratios (ORs) and 95% confidence intervals (CIs) were reported. All statistical analyses were conducted using IBM SPSS Statistics for Windows, Version 24 (Released 2016; IBM Corp., Armonk, New York) and R Version 4.3.2 (The Free Software Foundation, Boston, MA, USA). Additionally, the "mgcv" package was used to construct the GAMs. Statistical significance is set at a two-sided p-value of 0.05.

Ethical considerations

The study was carried out in compliance with the Declaration of Helsinki. The data used in this study were publicly available data from the NHANES database. The National Center for Health Statistics (NCHS) Ethics Review Board (ERB) examined and approved all the NHANES protocols, with all participants signing the informed consent.

## Results

A total of 3141 women were enrolled in this study, of whom 9.55% had depression symptoms. As shown in Table 1, depression symptoms were different in race, BMI, smoking status, drinking status, hypertension, and age at menarche. Additionally, participants with depression symptoms were more likely to have irregular menstruation and lower sleep duration (all P<0.05).

**Table 1:** General characteristics of the participants IQR: interquartile range; BMI: body mass index

Characteristics	Total (n = 3141)	Non-depression symptoms (n = 2841)	Depression symptoms (n = 300)	Mann-Whitney U test/chi-square test (z/χ^2^)	P-value
Age (years), median (IQR)	31 (24, 39)	31 (24, 39)	33 (23, 40)	-1.409	0.159
Race (n, %)				4.423	0.041
Non-Hispanic White	1036 (33.0)	914 (32.2)	122 (40.7)	-	-
Other	2105 (67.0)	1927 (67.8)	178 (59.3)	-	-
BMI (kg/m^2^), median (IQR)	27.8 (22.9, 33.7)	27.6 (22.8, 33.4)	29.4 (24.1, 36.1)	-3.446	0.001
BMI group (n, %)				2.419	0.096
Under & normal	1126 (35.8)	1041 (36.7)	85 (28.3)	-	-
Overweight	776 (24.7)	702 (24.7)	74 (24.7)	-	-
Obese	1239 (39.5)	1091 (38.4)	141 (47.0)	-	-
Smoking status (n, %)				50.071	< 0.001
Non-smoker	2319 (73.8)	2162 (76.1)	157 (52.3)	-	-
Former smoker	300 (9.6)	271 (9.5)	29 (9.7)	-	-
Current smoker	522 (16.6)	408 (14.4)	114 (38.0)	-	-
Drinking status (n, %)				4.690	0.012
Non-drinker	616 (19.6)	579 (20.4)	37 (12.3)	-	-
Former drinker	339 (10.8)	307 (10.8)	32(10.7)	-	-
Current drinker	2186 (69.6)	1955 (68.8)	231 (77.0)	-	-
Hypertension (n, %)				12.510	0.001
Yes	443 (14.1)	375 (13.2)	68 (22.7)	-	-
No	2698 (85.9)	2466 (86.8)	232 (77.3)	-	-
Diabetes (n, %)				3.002	0.090
Yes	172 (5.5)	145 (5.1)	27 (9.0)	-	-
No	2969 (94.5)	2696 (94.9)	273 (91.0)	-	-
Age at menarche (years), median (IQR)	12 (12, 13)	12 (12, 13)	12 (11, 13)	-3.320	0.001
Irregular menstruation				7.628	0.008
Yes	3 (9.6)	257 (9.0)	43 (14.3)	-	-
No	2841 (90.4)	2584 (90.1)	257 (85.7)	-	-
Sleep duration (hours), median (IQR)	7.5 (6.5, 8.0)	7.5 (6.5, 8.0)	7 (6, 8)	-3.515	< 0.001

Table 2 shows the relationship between irregular menstruation and sleep duration with depression symptoms. Sleep duration was associated with depression symptoms in three models (OR=0.848, (95% CI: 0.751-0.959); OR=0.864, (95% CI: 0.747-0.959); OR=0.872, (95% CI: 0.774-0.983), respectively), and those participants with irregular menstruation were more likely to have a higher prevalence of depression symptoms in three models (OR=1.679, (95% CI: 1.134-2.458); OR=1.679, (95% CI: 1.138-2.477); OR=1.595, (95% CI: 1.065-2.389), respectively).

**Table 2:** Relationship of irregular menstruation and sleep duration with depression symptoms Model 1: Unadjusted; Model 2: Adjusted for age, race, and BMI; Model 3: Adjusted for age, race, BMI, smoking status, drinking status, hypertension, and age at menarche. CI: confidence interval; OR: odds ratio; BMI: body mass index

Variables	Model 1			Model 2			Model 3		
	β-value	OR (95% CI)	P-value	β-value	OR (95% CI)	P-value	β-value	OR (95% CI)	P-value
Irregular menstruation									
No	-	1.000 (reference)	-	-	1.000 (reference)	-	-	1.000 (reference)	-
Yes	0.518	1.679 (1.134, 2.458)	0.011	0.518	1.679 (1.138, 2.477)	0.010	0.467	1.595 (1.065, 2.389)	0.025
Sleep duration	-0.164	0.848 (0.751, 0.959)	0.010	-0.167	0.864 (0.747, 0.959)	0.010	-0.137	0.872 (0.774, 0.983)	0.026

**Figure 2:**
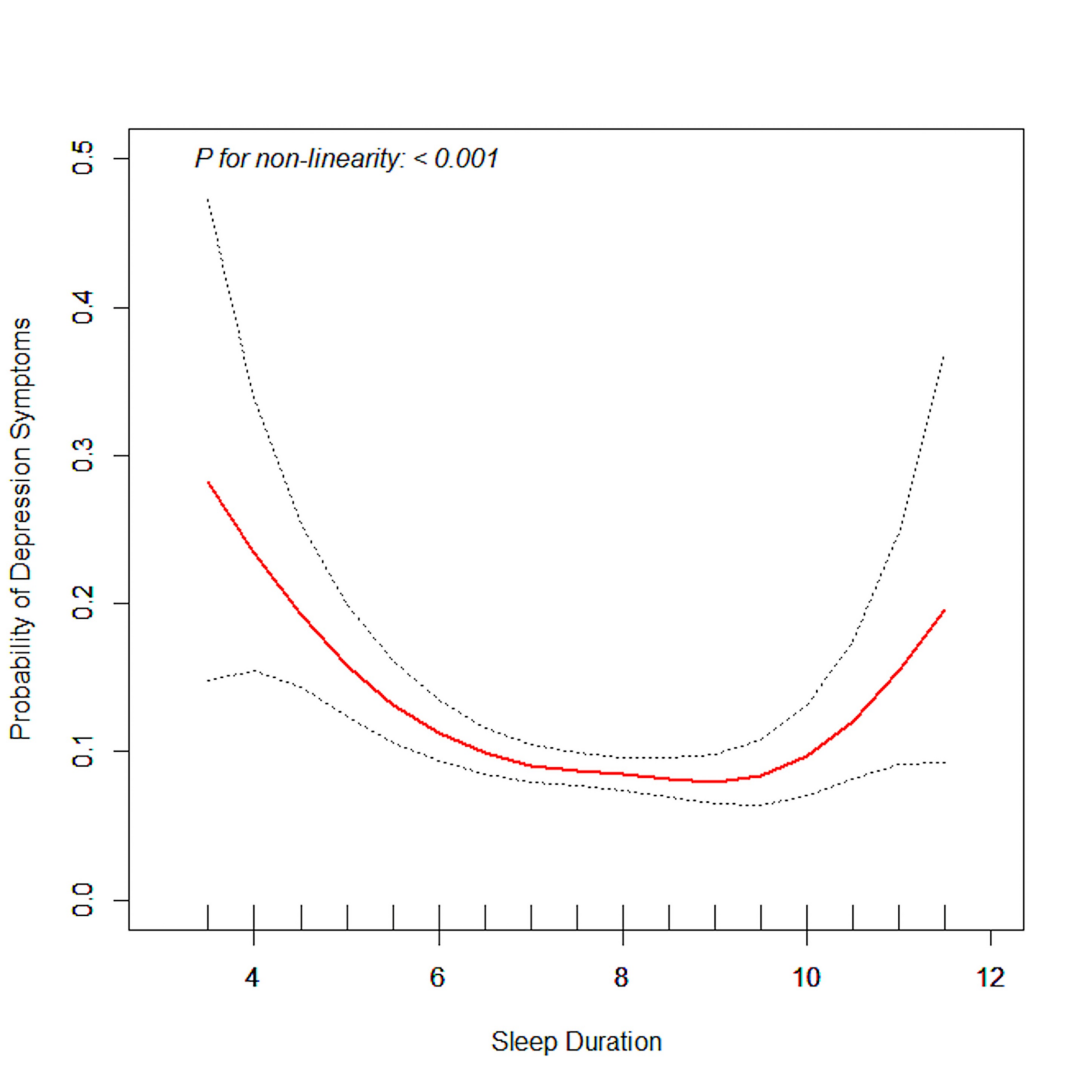
Association of sleep duration and depression symptoms

A U-shaped association was found between sleep duration and depression symptoms (Figure 2) with adjustments for all covariates. Threshold effect analysis demonstrated that the inflection point of sleep duration was nine hours. When the sleep duration was <9 hours, increased sleep duration was negatively associated with depression symptoms (OR=0.97, (95% CI: 0.96-0.98), P<0.001); each one-hour increase in sleep duration equaled a 3% decrease in the probability of depression symptoms. However, when the sleep duration was ≥9 hours, sleep duration was positively connected with depression symptoms (OR=1.06, (95% CI: 1.03-1.10), P<0.001). Each one-hour increase in sleep duration equaled a 6% increase in the predicted probability of depressive symptoms (Table 3).

**Table 3:** Threshold effect analysis of sleep duration on depression symptoms Adjusted for age, race, BMI, smoking status, drinking status, hypertension, and age at menarche. OR: odds ratio; CI: confidence interval; BMI: body mass index

Outcome	OR (95% CI)	P-value
Binary logistic regression model	0.98 (0.98, 0.99)	< 0.001
Two-piecewise logistic regression model	-	-
Sleep duration < 9 hours	0.97 (0.96, 0.98)	< 0.001
Sleep duration ≥ 9 hours	1.06 (1.03, 1.10)	< 0.001
Log-likelihood ratio test	-	< 0.001

An interaction was observed between irregular menstruation and sleep duration regarding depression symptoms after adjusting for all covariates (OR=0.736 (95% CI: 0.554-0.977), P=0.035). Based on the interaction, we focused on the relationship between irregular menstruation and depression symptoms stratified by sleep duration. For participants with sleep duration of <9 hours rather than ≥9 hours, irregular menstruation was positively correlated with depression symptoms in three models (OR=1.740, (95% CI: 1.193-2.537); OR=1.649, (95% CI: 1.121-2.427); OR=1.548, (95% CI: 1.040-2.304), respectively) (Table 4).

**Table 4:** Association between irregular menstruation and depression symptoms stratified by sleep duration Model 1: Unadjusted. Model 2: Adjusted for age, race, and BMI. Model 3: Adjusted for age, race, BMI, smoking status, drinking status, hypertension, and age at menarche. OR: odds ratio; CI: confidence interval; BMI: body mass index

Sleep duration	Irregular menstruation	Model 1			Model 2			Model 3		
		β-value	OR (95% CI)	P-value	β-value	OR (95% CI)	P-value	β-value	OR (95% CI)	P-value
< 9	No	-	1.000 (reference)	-	-	1.000 (reference)	-	-	1.000 (reference)	-
	Yes	0.554	1.740 (1.193, 2.537)	0.004	0.500	1.649 (1.121, 2.427)	0.011	0.437	1.548 (1.040, 2.304)	0.031
≥ 9	No	-	1.000 (reference)	-	-	-	-	-	-	-
	Yes	0.335	1.398 (0.567, 3.447)	0.467	0.129	1.138 (0.451, 2.869)	0.784	-0.039	0.962 (0.364, 2.543)	0.937

## Discussion

Irregular menstruation, a common complaint among women, is a key indicator of health. However, it gets very little attention. We found a higher prevalence of irregular menstruation compared to previous studies (14.3% vs. 11.2%/8.4%) [8,16]. Differences in participants and regions could potentially explain the reason. A prior study [17] has found that irregular menstruation was associated with mental health, which was consistent with our study. On one hand, the hypothalamic-pituitary axis (HPA) might have an impact on gonadotropin-releasing hormone (GnRH), and the dysregulation of GnRH predisposes women to depression [18]. On the other hand, some studies believed that there may exist a bidirectional relationship between irregular menstruation and depression symptoms; disorders in one can make an impact on the other, and women with depressed mood were more likely to have irregular menstruation [19]. Therefore, a longitudinal study should be designed to determine the causality between irregular menstruation and depression.

Sleep duration is an important quantitative indicator often used to evaluate sleep quality. Prior studies showed that sleep duration was associated with mental health [20], and our study aimed to examine the relationship between sleep duration and depression symptoms in women of childbearing age, which was the first time targeting this specific group. A U-shaped relationship between sleep duration and depression symptoms was found, which was consistent with a previous study [11]. We found that the prevalence of depression symptoms increased dramatically with extreme sleep duration, and the optimal sleep duration to reduce the prevalence of depression symptoms was nine hours in women of childbearing age. Both short and long sleep durations could increase the prevalence of depression symptoms.

Short sleep duration is one of the most common manifestations of sleep disorders, which is related to inflammation in the body. On the one hand, sleep disorders could lead to an increase in inflammation [21], and the elevated levels of inflammatory markers CRP and IL-6 could increase the prevalence of depression symptoms [22]. On the other hand, Eisenberger et al. found that inflammation in the body could activate brain sites that monitor positive or negative emotions and might increase depressive mood, which is more common in women [23]. In addition, inflammation might be one of the pathways linking sleep disorders and depression symptoms, and there may exist a bidirectional relationship between depression symptoms and sleep disorders [24]; a longitudinal study should be conducted.

We found that long sleep duration could also increase the prevalence of depression symptoms. Participants with long sleep durations may experience long periods of fragmented sleep and rapid eye movement sleep, which are associated with several negative health outcomes, including a decline in energy and vitality [25]. Furthermore, a prior study found that physical activity can improve the poor health conditions of adults, especially in terms of improving their mental health [26]. People with decreased energy and vitality were less likely to engage in physical exercise, which contributed to a higher prevalence of depression symptoms. Thus, encouraging women of childbearing age to ensure appropriate sleep duration through other means, such as participating in physical activities, may indirectly affect depressive symptoms.

Our study explored the interaction of irregular menstruation and sleep duration on depression symptoms in women of childbearing age for the first time, and an interaction was found. In the short sleep duration group, irregular menstruation was significantly associated with depression symptoms, while in the long sleep duration group, a relationship between irregular menstruation and depression symptoms was not found. Meers et al. found that women with unstable estrogen levels are more likely to experience sleep disruption and negative emotions; long sleep duration may relieve this poor sleep condition and directly regulate the impact of unstable estrogen on poor emotions [27]. Changes in estrogen levels lead women to prefer trait-based measures of pressure and ruminative thinking [28], and this risk may be exacerbated by the effects of sleep disorders [29], further increasing the prevalence of developing mental disorders. Therefore, more attention should be paid to women with short sleep duration and irregular menstruation, which might provide potential intervention targets for depression symptoms.

One of the strengths of our research was that we focused on the association of irregular menstruation and sleep duration with depression symptoms in women of childbearing age, which was the first time targeting this particular group. Second, threshold effect analysis was applied to analyze the relationship between sleep duration and depression symptoms. Lastly, we explored the interaction of irregular menstruation and sleep duration with depression symptoms. However, this study has many limitations. Our study was cross-sectional in design, and it is difficult to determine causality. It focused on women of childbearing age in the United States, and more research is needed to determine whether our findings are applicable to other countries and regions. In addition, the data regarding depression symptoms or irregular menstruation were not diagnosed by professional clinicians, and they were obtained using self-report questionnaires. The assessment of irregular menstruation and sleep duration was based solely on questions, and self-report bias might exist. The study did not measure many potential confounding variables, such as hormonal contraceptives and stress levels.

## Conclusions

The study focused on women of childbearing age and identified some key findings. Irregular menstruation was associated with an increased prevalence of depression symptoms in women of childbearing age. A U-shaped relationship between sleep duration and depression symptoms was found, and the cutoff point was nine hours in women of childbearing age. An interaction of irregular menstruation and sleep duration on depression symptoms was found in women of childbearing age. These findings may help women pay attention to their menstrual cycle and maintain proper sleep duration, which may help them implement targeted interventions for depression symptoms and enhance their quality of life.

## References

[1] Patel RB, Rao HR, Thakkar DV, Patel MR (2022). Comprehending the potential of metallic, lipid, and polymer-based nanocarriers for treatment and management of depression. Neurochem Int.

[2] Chunnan L, Shaomei S, Wannian L (2022). The association between sleep and depressive symptoms in US adults: data from the NHANES (2007-2014). Epidemiol Psychiatr Sci.

[3] Tang X, Lu Z, Hu D, Zhong X (2019). Influencing factors for prenatal Stress, anxiety and depression in early pregnancy among women in Chongqing, China. J Affect Disord.

[4] Morssinkhof MW, Lamers F, Hoogendoorn AW (2021). Oral contraceptives, depressive and insomnia symptoms in adult women with and without depression. Psychoneuroendocrinology.

[5] Foster C, Al-Zubeidi H (2018). Menstrual irregularities. Pediatr Ann.

[6] Kwak Y, Kim Y, Baek KA (2019). Prevalence of irregular menstruation according to socioeconomic status: a population-based nationwide cross-sectional study. PLoS One.

[7] Maurya P, Meher T, Muhammad T (2022). Relationship between depressive symptoms and self-reported menstrual irregularities during adolescence: evidence from UDAYA, 2016. BMC Public Health.

[8] Toffol E, Koponen P, Luoto R, Partonen T (2014). Pubertal timing, menstrual irregularity, and mental health: results of a population-based study. Arch Womens Ment Health.

[9] Hirshkowitz M, Whiton K, Albert SM (2015). National Sleep Foundation's sleep time duration recommendations: methodology and results summary. Sleep Health.

[10] Um YJ, Kim Y, Chang Y, Jung HS, Cho IY, Jeon SW, Ryu S (2023). Association of changes in sleep duration and quality with incidence of depression: a cohort study. J Affect Disord.

[11] Geoffroy PA, Tebeka S, Blanco C, Dubertret C, Le Strat Y (2020). Shorter and longer durations of sleep are associated with an increased twelve-month prevalence of psychiatric and substance use disorders: findings from a nationally representative survey of US adults (NESARC-III). J Psychiatr Res.

[12] Zhong W, Wang F, Chi L, Yang X, Yang Y, Wang Z (2022). Association between sleep duration and depression among the elderly population in China. Exp Aging Res.

[13] Arya S, Dwivedi AK, Alvarado L, Kupesic-Plavsic S (2020). Exposure of U.S. population to endocrine disruptive chemicals (Parabens, Benzophenone-3, Bisphenol-A and Triclosan) and their associations with female infertility. Environ Pollut.

[14] Sun M, Wang L, Wang X (2023). Interaction between sleep quality and dietary inflammation on frailty: NHANES 2005-2008. Food Funct.

[15] Wang X, Chen Z, Cheng D (2023). Association between urinary metabolites of volatile organic compounds and cardiovascular disease in the general population from NHANES 2011-2018. Ecotoxicol Environ Saf.

[16] Fielder S, Nickkho-Amiry M, Seif MW (2023). Obesity and menstrual disorders. Best Pract Res Clin Obstet Gynaecol.

[17] Klusmann H, Kapp C, Engel S, Schumacher T, Bücklein E, Knaevelsrud C, Schumacher S (2024). Higher depressive symptoms in irregular menstrual cycles: converging evidence from cross-sectional and prospective assessments. Psychopathology.

[18] Klimes-Dougan B, Begnel E, Almy B, Thai M, Schreiner MW, Cullen KR (2019). Hypothalamic-pituitary-adrenal axis dysregulation in depressed adolescents with non-suicidal self-injury. Psychoneuroendocrinology.

[19] Padda J, Khalid K, Hitawala G (2021). Depression and its effect on the menstrual cycle. Cureus.

[20] Vestergaard CL, Skogen JC, Hysing M, Harvey AG, Vedaa Ø, Sivertsen B (2024). Sleep duration and mental health in young adults. Sleep Med.

[21] Irwin MR, Olmstead R, Carroll JE (2016). Sleep disturbance, sleep duration, and inflammation: a systematic review and meta-analysis of cohort studies and experimental sleep deprivation. Biol Psychiatry.

[22] Irwin MR, Opp MR (2017). Sleep health: reciprocal regulation of sleep and innate immunity. Neuropsychopharmacology.

[23] Eisenberger NI, Inagaki TK, Mashal NM, Irwin MR (2010). Inflammation and social experience: an inflammatory challenge induces feelings of social disconnection in addition to depressed mood. Brain Behav Immun.

[24] Cho HJ, Eisenberger NI, Olmstead R, Breen EC, Irwin MR (2016). Preexisting mild sleep disturbance as a vulnerability factor for inflammation-induced depressed mood: a human experimental study. Transl Psychiatry.

[25] Youngstedt SD, Kripke DF (2004). Long sleep and mortality: rationale for sleep restriction. Sleep Med Rev.

[26] Ge Y, Xin S, Luan D, Zou Z, Liu M, Bai X, Gao Q (2019). Association of physical activity, sedentary time, and sleep duration on the health-related quality of life of college students in Northeast China. Health Qual Life Outcomes.

[27] Meers JM, Bower J, Nowakowski S, Alfano C (2024). Interaction of sleep and emotion across the menstrual cycle. J Sleep Res.

[28] Kappen M, Raeymakers S, Weyers S, Vanderhasselt MA (2022). Stress and rumination in premenstrual syndrome (PMS): identifying stable and menstrual cycle-related differences in PMS symptom severity. J Affect Disord.

[29] Meers JM, Bower JL, Alfano CA (2020). Poor sleep and emotion dysregulation mediate the association between depressive and premenstrual symptoms in young adult women. Arch Womens Ment Health.

